# 2,10-Dibromo-6-isobutyl-6-methyl­dibenzo[*d*,*f*][1,3]dioxepine

**DOI:** 10.1107/S1600536808011665

**Published:** 2008-04-26

**Authors:** Hai-Quan Zhang, Bao-Li, Guang-Di Yang, Yu-Guang Ma

**Affiliations:** a State Key Laboratory of Metastable Materials Science and Technology, Yanshan University, Qinhuangdao 066004, People’s Republic of China; b State Key Laboratory of Supramolecular Structure and Materials, Jilin University, Changchun 130012, People’s Republic of China

## Abstract

In the crystal structure of the title compound, C_18_H_18_Br_2_O_2_, the two benzene rings of the bridged biphenyl unit are twisted by 38.0 (1)°.

## Related literature

For the synthesis of the title compound, see: Zhang *et al*. (2003 ▶).
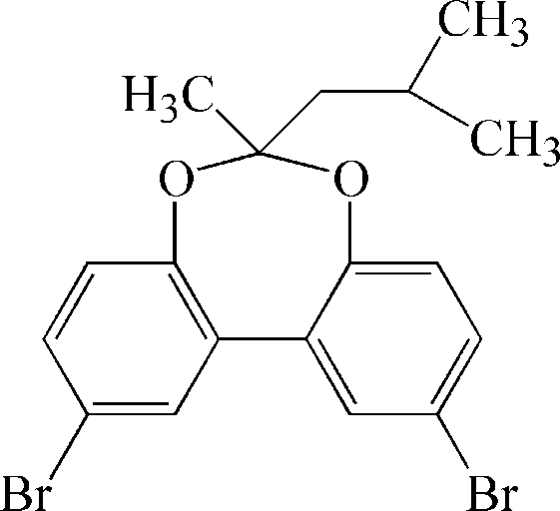


## Experimental

### 

#### Crystal data

C_18_H_18_Br_2_O_2_
*M* *_r_* = 426.14
Monoclinic, 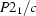

*a* = 8.3372 (9) Å
*b* = 19.362 (2) Å
*c* = 10.8066 (16) Å
β = 100.803 (5)°
*V* = 1713.5 (4) Å^3^
*Z* = 4
Mo *K*α radiation
μ = 4.74 mm^−1^
*T* = 193 (2) K
0.13 × 0.12 × 0.12 mm

#### Data collection

Rigaku R-AXIS RAPID diffractometer
Absorption correction: multi-scan (*ABSCOR*; Higashi, 1995 ▶) *T*_min_ = 0.587, *T*_max_ = 0.607 (expected range = 0.547–0.567)
7425 measured reflections
3924 independent reflections
2495 reflections with *I* > 2σ(*I*)
*R*_int_ = 0.028

#### Refinement

*R*[*F* ^2^ > 2σ(*F* ^2^)] = 0.028
*wR*(*F* ^2^) = 0.052
*S* = 0.85
3924 reflections
202 parameters
H-atom parameters constrained
Δρ_max_ = 0.41 e Å^−3^
Δρ_min_ = −0.51 e Å^−3^

### 

Data collection: *RAPID-AUTO* (Rigaku, 1998 ▶); cell refinement: *RAPID-AUTO*; data reduction: *CrystalStructure* (Rigaku/MSC and Rigaku, 2002 ▶); program(s) used to solve structure: *SHELXS97* (Sheldrick, 2008 ▶); program(s) used to refine structure: *SHELXL97* (Sheldrick, 2008 ▶); molecular graphics: *PLATON* (Spek, 2003 ▶); software used to prepare material for publication: *SHELXL97* .

## Supplementary Material

Crystal structure: contains datablocks global, I. DOI: 10.1107/S1600536808011665/nc2099sup1.cif

Structure factors: contains datablocks I. DOI: 10.1107/S1600536808011665/nc2099Isup2.hkl

Additional supplementary materials:  crystallographic information; 3D view; checkCIF report

## Figures and Tables

**Fig. 1.**
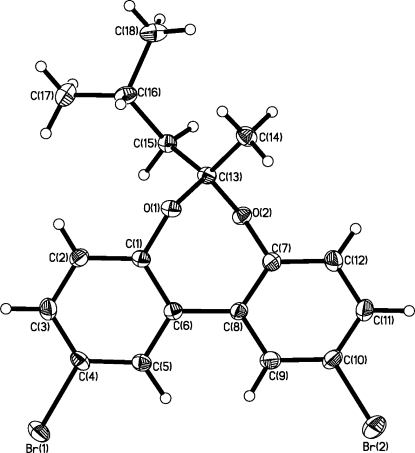
Crystal structure of the title compound with the atom-labelling scheme. Displacement ellipsoids are drawn at the 30% probability level. H atoms are represented as small spheres of arbitrary radii.

## supplementary crystallographic information

### Comment

Recently we have reported on the synthesis of the title compound 2,10-Dibromo -6-isobutyl-6-methyl-Dibenzo [d,f][1,3] Dioxepine (Zhang *et al.* 2003). Herein we present the crysal structure of this compound. In the crystal structure of the title compound the two benzene rings of the bridged biphenyl unit are twisted by 38.0 (1)° and the 7-membered ring is in a boat conformation.

### Experimental

The title compound was synthesized as described previously (Zhang *et al.* 2003). Single crystals were obtained by slow evaporation of the solvent from a methanol solution at room temperature.

### Refinement

All H atoms were positioned with idealized geometry and refined isotropic with *U*_iso_(H) = 1.5*U*_eq_(C) for methyl and *U*_iso_(H) = 1.2*U*_eq_(C)for all other H atoms using a riding model with C—H = 0.97 Å for methyl and C—H = 0.93 Å for all other H atoms.

### Crystal data

**Table:**

C_18_H_18_Br_2_O_2_	*F*_000_ = 848
*M**_r_* = 426.14	*D*_x_ = 1.652 Mg m^−^^3^
Monoclinic, *P*2_1_/*c*	Mo *K*α radiation λ = 0.71073 Å
Hall symbol: -P 2ybc	Cell parameters from 12167 reflections
*a* = 8.3372 (9) Å	θ = 2.1–54.9º
*b* = 19.362 (2) Å	µ = 4.74 mm^−^^1^
*c* = 10.8066 (16) Å	*T* = 193 (2) K
β = 100.803 (5)º	Block, colorless
*V* = 1713.5 (4) Å^3^	0.13 × 0.12 × 0.12 mm
*Z* = 4	

### Data collection

**Table:**

Rigaku R-AXIS RAPID diffractometer	3924 independent reflections
Radiation source: fine-focus sealed tube	2495 reflections with *I* > 2σ(*I*)
Monochromator: graphite	*R*_int_ = 0.028
*T* = 193(2) K	θ_max_ = 27.5º
ω scans	θ_min_ = 2.1º
Absorption correction: multi-scan(ABSCOR; Higashi, 1995)	*h* = −10→10
*T*_min_ = 0.587, *T*_max_ = 0.607	*k* = −25→25
7425 measured reflections	*l* = −14→14

### Refinement

**Table:**

Refinement on *F*^2^	Secondary atom site location: difference Fourier map
Least-squares matrix: full	Hydrogen site location: inferred from neighbouring sites
*R*[*F*^2^ > 2σ(*F*^2^)] = 0.028	H-atom parameters constrained
*wR*(*F*^2^) = 0.052	*w* = 1/[σ^2^(*F*_o_^2^) + (0.0206*P*)^2^] where *P* = (*F*_o_^2^ + 2*F*_c_^2^)/3
*S* = 0.85	(Δ/σ)_max_ = 0.001
3924 reflections	Δρ_max_ = 0.41 e Å^−^^3^
202 parameters	Δρ_min_ = −0.51 e Å^−^^3^
Primary atom site location: structure-invariant direct methods	Extinction correction: none

### Special details

**Table:**

Geometry. All e.s.d.'s (except the e.s.d. in the dihedral angle between two l.s. planes) are estimated using the full covariance matrix. The cell e.s.d.'s are taken into account individually in the estimation of e.s.d.'s in distances, angles and torsion angles; correlations between e.s.d.'s in cell parameters are only used when they are defined by crystal symmetry. An approximate (isotropic) treatment of cell e.s.d.'s is used for estimating e.s.d.'s involving l.s. planes.
Refinement. Refinement of *F*^2^ against ALL reflections. The weighted *R*-factor *wR* and goodness of fit *S* are based on *F*^2^, conventional *R*-factors *R* are based on *F*, with *F* set to zero for negative *F*^2^. The threshold expression of *F*^2^ > σ(*F*^2^) is used only for calculating *R*-factors(gt) *etc*. and is not relevant to the choice of reflections for refinement. *R*-factors based on *F*^2^ are statistically about twice as large as those based on *F*, and *R*- factors based on ALL data will be even larger.

### Fractional atomic coordinates and isotropic or equivalent isotropic displacement parameters (Å^2^)

**Table:**

	*x*	*y*	*z*	*U*_iso_*/*U*_eq_	
Br1	1.32353 (3)	0.596022 (13)	0.68875 (3)	0.03946 (9)	
Br2	1.32322 (4)	0.994048 (14)	0.80855 (4)	0.06258 (12)	
C1	1.0296 (3)	0.70180 (11)	0.9608 (2)	0.0219 (5)	
C2	1.0540 (3)	0.63147 (11)	0.9678 (2)	0.0273 (6)	
H2	1.0100	0.6051	1.0276	0.033*	
C3	1.1424 (3)	0.59896 (11)	0.8880 (2)	0.0294 (6)	
H3	1.1596	0.5504	0.8921	0.035*	
C4	1.2052 (3)	0.63899 (12)	0.8022 (2)	0.0264 (6)	
C5	1.1833 (3)	0.70927 (11)	0.7948 (2)	0.0238 (5)	
H5	1.2283	0.7354	0.7353	0.029*	
C6	1.0946 (3)	0.74206 (11)	0.8752 (2)	0.0220 (5)	
C7	0.9182 (3)	0.84461 (11)	0.8840 (2)	0.0240 (5)	
C8	1.0681 (3)	0.81720 (11)	0.8698 (2)	0.0226 (5)	
C9	1.1879 (3)	0.86301 (12)	0.8475 (2)	0.0305 (6)	
H9	1.2914	0.8460	0.8370	0.037*	
C10	1.1573 (3)	0.93303 (12)	0.8403 (2)	0.0329 (7)	
C11	1.0099 (3)	0.95998 (11)	0.8559 (2)	0.0333 (6)	
H11	0.9914	1.0084	0.8517	0.040*	
C12	0.8886 (3)	0.91495 (11)	0.8779 (2)	0.0297 (6)	
H12	0.7856	0.9324	0.8887	0.036*	
C13	0.7930 (3)	0.76216 (11)	1.0048 (2)	0.0231 (5)	
C14	0.7645 (3)	0.81026 (11)	1.1085 (2)	0.0304 (6)	
H14A	0.7726	0.7843	1.1873	0.046*	
H14B	0.6554	0.8308	1.0860	0.046*	
H14C	0.8469	0.8470	1.1195	0.046*	
C15	0.6684 (3)	0.70529 (11)	0.9684 (2)	0.0239 (5)	
H15A	0.7027	0.6767	0.9020	0.029*	
H15B	0.5622	0.7267	0.9316	0.029*	
C16	0.6434 (3)	0.65766 (12)	1.0774 (2)	0.0271 (6)	
H16	0.7452	0.6592	1.1431	0.032*	
C17	0.6163 (3)	0.58315 (12)	1.0329 (2)	0.0404 (7)	
H17A	0.6030	0.5538	1.1041	0.061*	
H17B	0.7106	0.5673	0.9984	0.061*	
H17C	0.5177	0.5804	0.9674	0.061*	
C18	0.5010 (3)	0.68121 (13)	1.1386 (2)	0.0414 (7)	
H18A	0.3989	0.6784	1.0767	0.062*	
H18B	0.5193	0.7290	1.1677	0.062*	
H18C	0.4938	0.6512	1.2104	0.062*	
O1	0.95321 (18)	0.73412 (7)	1.04861 (13)	0.0232 (4)	
O2	0.78958 (17)	0.79979 (7)	0.88965 (14)	0.0243 (4)	

### Atomic displacement parameters (Å^2^)

**Table:**

	*U*^11^	*U*^22^	*U*^33^	*U*^12^	*U*^13^	*U*^23^
Br1	0.03994 (16)	0.03630 (15)	0.04601 (17)	0.00641 (13)	0.01797 (13)	−0.00815 (14)
Br2	0.0675 (2)	0.03245 (16)	0.1003 (3)	−0.01841 (15)	0.0480 (2)	−0.00512 (17)
C1	0.0173 (13)	0.0274 (13)	0.0206 (13)	0.0013 (10)	0.0024 (11)	0.0006 (10)
C2	0.0242 (13)	0.0273 (13)	0.0309 (15)	0.0009 (11)	0.0066 (12)	0.0070 (11)
C3	0.0267 (13)	0.0213 (12)	0.0391 (16)	0.0033 (11)	0.0029 (12)	0.0019 (12)
C4	0.0170 (13)	0.0304 (13)	0.0316 (15)	0.0042 (10)	0.0039 (12)	−0.0069 (11)
C5	0.0198 (12)	0.0276 (13)	0.0243 (14)	0.0006 (10)	0.0050 (11)	0.0011 (11)
C6	0.0178 (13)	0.0247 (12)	0.0225 (13)	−0.0005 (10)	0.0016 (11)	−0.0007 (10)
C7	0.0244 (14)	0.0260 (13)	0.0212 (14)	−0.0015 (10)	0.0030 (11)	−0.0009 (10)
C8	0.0283 (13)	0.0207 (11)	0.0205 (13)	−0.0008 (10)	0.0083 (11)	−0.0004 (10)
C9	0.0306 (15)	0.0298 (14)	0.0338 (17)	−0.0028 (11)	0.0131 (14)	−0.0036 (11)
C10	0.0423 (16)	0.0252 (13)	0.0362 (17)	−0.0089 (12)	0.0203 (14)	−0.0007 (11)
C11	0.0458 (18)	0.0205 (12)	0.0354 (16)	0.0023 (12)	0.0122 (14)	0.0013 (11)
C12	0.0313 (14)	0.0279 (14)	0.0303 (15)	0.0064 (11)	0.0067 (12)	0.0034 (11)
C13	0.0245 (13)	0.0279 (12)	0.0167 (13)	0.0044 (11)	0.0032 (11)	0.0033 (10)
C14	0.0294 (14)	0.0320 (14)	0.0304 (16)	0.0018 (12)	0.0073 (12)	−0.0052 (12)
C15	0.0207 (13)	0.0280 (12)	0.0222 (13)	0.0017 (10)	0.0020 (11)	−0.0004 (11)
C16	0.0215 (13)	0.0352 (14)	0.0237 (14)	−0.0039 (11)	0.0025 (11)	0.0023 (11)
C17	0.0423 (17)	0.0333 (15)	0.0468 (18)	−0.0035 (13)	0.0116 (14)	0.0074 (13)
C18	0.0331 (16)	0.0539 (17)	0.0408 (18)	−0.0094 (13)	0.0163 (14)	−0.0011 (14)
O1	0.0193 (9)	0.0305 (9)	0.0197 (9)	0.0023 (7)	0.0033 (7)	0.0001 (7)
O2	0.0226 (9)	0.0266 (9)	0.0230 (9)	−0.0009 (7)	0.0028 (8)	0.0043 (7)

### Geometric parameters (Å, °)

**Table:**

Br1—C4	1.903 (2)	C11—H11	0.9500
Br2—C10	1.898 (2)	C12—H12	0.9500
C1—C2	1.377 (3)	C13—O1	1.437 (3)
C1—O1	1.387 (2)	C13—O2	1.438 (2)
C1—C6	1.395 (3)	C13—C14	1.510 (3)
C2—C3	1.386 (3)	C13—C15	1.515 (3)
C2—H2	0.9500	C14—H14A	0.9800
C3—C4	1.384 (3)	C14—H14B	0.9800
C3—H3	0.9500	C14—H14C	0.9800
C4—C5	1.373 (3)	C15—C16	1.541 (3)
C5—C6	1.394 (3)	C15—H15A	0.9900
C5—H5	0.9500	C15—H15B	0.9900
C6—C8	1.471 (3)	C16—C17	1.524 (3)
C7—C12	1.384 (3)	C16—C18	1.532 (3)
C7—O2	1.390 (2)	C16—H16	1.0000
C7—C8	1.393 (3)	C17—H17A	0.9800
C8—C9	1.390 (3)	C17—H17B	0.9800
C9—C10	1.379 (3)	C17—H17C	0.9800
C9—H9	0.9500	C18—H18A	0.9800
C10—C11	1.375 (3)	C18—H18B	0.9800
C11—C12	1.390 (3)	C18—H18C	0.9800
			
C2—C1—O1	119.39 (18)	O1—C13—C14	104.76 (18)
C2—C1—C6	121.15 (19)	O2—C13—C14	110.46 (17)
O1—C1—C6	119.15 (19)	O1—C13—C15	111.15 (17)
C1—C2—C3	120.3 (2)	O2—C13—C15	103.98 (18)
C1—C2—H2	119.8	C14—C13—C15	116.49 (17)
C3—C2—H2	119.8	C13—C14—H14A	109.5
C4—C3—C2	118.3 (2)	C13—C14—H14B	109.5
C4—C3—H3	120.8	H14A—C14—H14B	109.5
C2—C3—H3	120.8	C13—C14—H14C	109.5
C5—C4—C3	122.06 (19)	H14A—C14—H14C	109.5
C5—C4—Br1	118.37 (16)	H14B—C14—H14C	109.5
C3—C4—Br1	119.57 (16)	C13—C15—C16	114.87 (19)
C4—C5—C6	119.70 (19)	C13—C15—H15A	108.6
C4—C5—H5	120.2	C16—C15—H15A	108.6
C6—C5—H5	120.2	C13—C15—H15B	108.6
C5—C6—C1	118.4 (2)	C16—C15—H15B	108.6
C5—C6—C8	121.27 (18)	H15A—C15—H15B	107.5
C1—C6—C8	120.31 (18)	C17—C16—C18	109.68 (19)
C12—C7—O2	119.0 (2)	C17—C16—C15	111.01 (19)
C12—C7—C8	121.7 (2)	C18—C16—C15	112.25 (19)
O2—C7—C8	118.87 (18)	C17—C16—H16	107.9
C9—C8—C7	117.69 (19)	C18—C16—H16	107.9
C9—C8—C6	122.05 (19)	C15—C16—H16	107.9
C7—C8—C6	120.24 (18)	C16—C17—H17A	109.5
C10—C9—C8	120.4 (2)	C16—C17—H17B	109.5
C10—C9—H9	119.8	H17A—C17—H17B	109.5
C8—C9—H9	119.8	C16—C17—H17C	109.5
C11—C10—C9	121.8 (2)	H17A—C17—H17C	109.5
C11—C10—Br2	118.97 (17)	H17B—C17—H17C	109.5
C9—C10—Br2	119.27 (18)	C16—C18—H18A	109.5
C10—C11—C12	118.6 (2)	C16—C18—H18B	109.5
C10—C11—H11	120.7	H18A—C18—H18B	109.5
C12—C11—H11	120.7	C16—C18—H18C	109.5
C7—C12—C11	119.8 (2)	H18A—C18—H18C	109.5
C7—C12—H12	120.1	H18B—C18—H18C	109.5
C11—C12—H12	120.1	C1—O1—C13	117.59 (17)
O1—C13—O2	110.04 (15)	C7—O2—C13	117.64 (17)
			
O1—C1—C2—C3	−174.4 (2)	C8—C9—C10—C11	0.6 (4)
C6—C1—C2—C3	−0.9 (4)	C8—C9—C10—Br2	−179.60 (18)
C1—C2—C3—C4	0.1 (4)	C9—C10—C11—C12	−0.8 (4)
C2—C3—C4—C5	0.5 (4)	Br2—C10—C11—C12	179.40 (19)
C2—C3—C4—Br1	−178.75 (18)	O2—C7—C12—C11	−171.7 (2)
C3—C4—C5—C6	−0.4 (4)	C8—C7—C12—C11	0.6 (4)
Br1—C4—C5—C6	178.88 (17)	C10—C11—C12—C7	0.2 (4)
C4—C5—C6—C1	−0.4 (3)	O1—C13—C15—C16	63.4 (2)
C4—C5—C6—C8	−179.8 (2)	O2—C13—C15—C16	−178.25 (16)
C2—C1—C6—C5	1.0 (4)	C14—C13—C15—C16	−56.5 (3)
O1—C1—C6—C5	174.5 (2)	C13—C15—C16—C17	−142.7 (2)
C2—C1—C6—C8	−179.6 (2)	C13—C15—C16—C18	94.2 (2)
O1—C1—C6—C8	−6.0 (3)	C2—C1—O1—C13	−111.5 (2)
C12—C7—C8—C9	−0.8 (4)	C6—C1—O1—C13	74.8 (3)
O2—C7—C8—C9	171.5 (2)	O2—C13—O1—C1	−45.4 (2)
C12—C7—C8—C6	−179.2 (2)	C14—C13—O1—C1	−164.10 (17)
O2—C7—C8—C6	−6.8 (3)	C15—C13—O1—C1	69.3 (2)
C5—C6—C8—C9	−37.5 (4)	C12—C7—O2—C13	−112.0 (2)
C1—C6—C8—C9	143.1 (2)	C8—C7—O2—C13	75.5 (2)
C5—C6—C8—C7	140.8 (2)	O1—C13—O2—C7	−45.3 (2)
C1—C6—C8—C7	−38.6 (3)	C14—C13—O2—C7	69.9 (2)
C7—C8—C9—C10	0.2 (4)	C15—C13—O2—C7	−164.38 (16)
C6—C8—C9—C10	178.5 (2)		
